# Quantum Transport in Mesoscopic Systems

**DOI:** 10.3390/e22090977

**Published:** 2020-09-01

**Authors:** David Sánchez, Michael Moskalets

**Affiliations:** 1 Institute for Cross-Disciplinary Physics and Complex Systems IFISC (UIB-CSIC), E-07122 Palma de Mallorca, Spain; 2 Department of Metal and Semiconductor Physics, National Technical University “Kharkiv Polytechnic Institute”, 61002 Kharkiv, Ukraine

**Keywords:** quantum transport, mesoscopic systems, nanophysics, quantum thermodynamics, quantum noise, quantum pumping, Kondo effect, thermoelectrics, heat transport

Mesoscopic physics has become a mature field. Its theoretical foundations and main models were established in the last two decades of the past century [1,2]. Ever since, quantum transport techniques have served as an excellent tool to understand the intriguing properties of charge carriers in nanoscale conductors [3,4]. However, in the last few years, the number of applications has grown so quickly that even experts find it difficult to stay updated with the recent advancements. The goal of the present special issue is to give a current snapshot of the field by means of a collection of review papers and research works that discuss the hottest theoretical questions and experimental results.

While the average current was the focus of early studies, the interest has gradually shifted to time-resolved transport. The motivation is partly due to new devices, such as single-electron emitters, which are able to inject quantized current pulses onto a Fermi sea for the investigation of inelastic and interaction effects upon electronic collisions. This is the subject of the review paper by Filippone et al. [5], in which Fermi liquid theories are employed to analyze strong correlations (Coulomb interactions) in the out-of-equilibrium dynamics of mesoscopic capacitors, a type of mesoscopic system whose response is purely dynamical. Here, dynamics is enforced via a time-dependent potential applied to a nearby gate. Under certain circumstances, the interplay of this potential and Coulomb interactions can lead to fractionalization effects in a single-electron transfer (quantized pumping). Chen and Zhu [6] find quantum pumping for a double-barrier system in the adiabatic limit. The novelty lies in their consideration of Dirac–Weyl quasiparticles. Tokura [7] also consider slow potentials, but the system is now an interferometer that allows not only for Aharanov–Bohm phases but also for spin-dependent shifts, due to both Rashba and Dresselhaus spin-orbit couplings. Meanwhile, Hashimoto and Uchiyama [8] tackle the nonadiabatic regime and present a complete analysis of the pumped charge, spin, and energy induced by temperature modulations in the attached reservoirs. A particularly useful approach that deals with this kind of problems is based on generalized master rate equations. Moldoveanu, Manolescu, and Gumundsson [9] illustrate the power of this method for a hybrid quantum-dot system that hosts both electronic and bosonic degrees of freedom. Among other things, they solve the master equations, including many-body effects in the transient response to time-dependent signals applied at the contact regions. Dynamically driven quantum devices are also suitable systems for testing alternative theoretical formulations. An example is the work of Pandey et al. [10], in which the Bohmian quantum theory is utilized to elucidate the role of non-Markovian conditions in graphene probed at very high frequencies.

In the recent cross-fertilization between thermodynamics and quantum physics, mesoscopic systems play a pivotal contribution. In their review article, Ansari, van Steensel, and Nazarov [11] connect information-theoretic concepts with the evaluation of entropy in quantum systems. They illustrate their discussion by calculating the entropy of various quantum heat engines. Quantum point contacts are prototypical mesoscopic devices that can precisely work as heat engines. It is, therefore, of utmost importance to understand their maximum generated power, as discussed by Kheradsoud et al. [12]. Interestingly, they find that power, efficiency, and fluctuations are bounded by thermodynamic uncertainty relations. Additionally, Bustos, Marún, and Calvo [13] introduce mechanical degrees of freedom to analyze the dynamics of quantum motors built, e.g., from double quantum dots coupled to rotors. A Langevin approach allows them to generically describe both motors and pumps out of equilibrium, which are relevant for quantum refrigeration setups. Remarkably, some of the well-established results in linear response (Onsager reciprocity, fluctuation-dissipation relations) also hold far from equilibrium. Maisel and López [14] demonstrate, with a capacitively coupled doubled quantum dot system, that it is possible to find bias configurations that lead to stalling currents, around which the above results were verified.

Quantum conductors constitute excellent platforms for the measurement and manipulation of thermal gradients and currents while keeping the quantum character of energy carriers. Biele and D’Agosta [15] review the standard theoretical approaches to quantum thermal transport (Landauer–Büttiker formalism and Boltzmann equation), pointing to their strengths and limitations. To overcome the latter, they discuss advanced methods, such as time-dependent density functional theory, the nonequilibrium Green’s functions approach, and density-matrix formulations. Atomistic computations are reviewed by Medrano Sandonas et al. [16] in the context of nanophononics. Clearly, phonons should be taken into account in any general description of heat transport in nanodevices, especially in molecular junctions. A density-functional tight-binding module specifically designed to deal with phonon transport is able to compute the phonon conductivity of molecules sandwiched between thermally biased metallic contacts. Perroni and Cataudella [17] consider the case of a fullerene and study the combined influence of vibrations and Coulomb interactions in the thermoelectric transport through the molecule.

In confined mesoscopic systems, electron–electron interactions can lead to strong correlations visible in transport measurements. A celebrated phenomenon is the Kondo effect, where the unpaired spin of an electron localized inside a quantum dot forms, at a low temperature, a many-body singlet with the spin density arising from conduction electrons that propagate in the leads attached to the dot. Tettamanzi [18] review the Kondo and the Kondo–Fano effects in silicon nanostructures, taking into account correlations between pseudospins belonging to different degeneracy points in the conduction bands. Simultaneous fluctuations in both the spin and pseudospin degrees of freedom give rise to higher symmetry Kondo states. Lee, Dong, and Lei [19] propose a multiterminal setup comprising a quantum dot attached to two ferromagnetic contacts and one superconducting lead, with the aim of assessing both local and nonlocal conductances within a slave-boson mean-field approximation. Their main finding is a competition between the superconductivity proximity effect, Kondo correlations, and spin polarizations that could be analyzed with a careful study of the conductance.

We began this Editorial with an emphasis on time-dependent currents. We would like to finish our presentation with a somewhat related quantity, namely, the noise, since current fluctuations are defined from time correlators. Bulka and Luczak [20] analyze the electric current noise in a ring structure that supports persistent currents. When the ring is pierced by an external magnetic field, the interference pattern is affected by the Aharonov–Bohm effect and this is reflected in the noise as a function of the flux. In mesoscopic conductors, not only charge, but also heat fluctuations, are significant. This leads to heat and mixed charge-heat correlators, as illustrated by Ronetti et al. [21] for a harmonically driven quantum Hall bar. The system shows quasiparticle excitations of fractional charge (Laughlin states), and it is demonstrated that the mixed noise differs for integer and fractional filling factors. Finally, current–current correlations can provide us with valuable information about the electronic traversal time through mesoscopic constrictions. Ridley, Sentef, and Tuovinen [22] calculate the cross-correlations of graphene nanoribbons and find that the sample disorder increased the traversal time.

Overall, these papers represent an outstanding perspective of current research in nanophysics. They show that the field is actively developing and alive with problems that are interesting to a great variety of physicists, whose concerns range from condensed matter to quantum information and thermodynamics. There is still plenty of room at the bottom, which implies fruitful opportunities in the near future.
